# Incidence and Risk Factors for Intraoperative Complications in Resident-performed Phacoemulsification Surgery

**DOI:** 10.18502/jovr.v20.14823

**Published:** 2025-07-21

**Authors:** Hossein Mohammad-Rabei, Homayoun Nikkhah, Zhale Rajavi, Mohammadmehdi Hatami, Mohammadsadegh Haghparast, Hamed Esfandiari

**Affiliations:** ^1^Ophthalmic Research Center, Research Institute for Ophthalmology and Vision Science, Shahid Beheshti University of Medical Sciences, Tehran, Iran; ^2^Department of Ophthalmology, Torfeh Hospital, Shahid Beheshti University of Medical Sciences, Tehran, Iran; ^3^Negah Aref Ophthalmic Research Center, Negah Eye Hospital, Tehran, Iran; ^4^Department of Ophthalmology, Olmsted Medical Center, Rochester, Minnesota, USA

**Keywords:** Intraoperative Complication, Phacoemulsification, Posterior Capsular Rupture, Resident Training Level, Vitreous Loss

## Abstract

**Purpose:**

To investigate the incidence and risk factors of intraoperative complications during phacoemulsification surgery performed by ophthalmology residents at an academic training center.

**Methods:**

This comparative case series included 1121 eyes of 821 patients that underwent phacoemulsification cataract surgery by ophthalmology residents from March 2020 to March 2021. Patients' medical records were reviewed for demographics, systemic and ophthalmic comorbidities, biometric data, surgical details, anesthesia type, residents' training year, and the occurrence of any intraoperative complications. Multivariate models were used to identify potential risk factors for intraoperative complications.

**Results:**

Intraoperative complications were observed in 63 eyes (5.6%). From the most to the least frequent, they included posterior capsular rupture with vitreous loss in 29 eyes (2.6%), anterior capsular tear in 21 eyes (1.9%), nucleus drop in 8 eyes (0.7%), zonular dehiscence in 3 eyes (0.3%), and posterior capsular rupture without vitreous loss in 2 eyes (0.2%). On univariate analysis, mature or brunescent cataracts (OR = 3.096, *P* = 0.031), residents' training year (OR = 2.238, *P* = 0.017), and pseudoexfoliation (OR = 2.247, *P* = 0.049) were associated with vitreous loss. Multivariate data analysis indicated that mature or brunescent cataracts (OR = 4.046, *P* = 0.009) and residents' training year (OR = 3.238, *P* = 0.005) were independent risk factors for vitreous loss.

**Conclusion:**

We observed a higher rate of intraoperative complications in eyes with mature or brunescent cataracts or in procedures performed by less experienced residents. Proper case selection and direct attending supervision are crucial in preventing intraoperative complications at ophthalmology training centers.

##  INTRODUCTION

Cataracts remain the leading cause of reversible blindness worldwide. In 2020, approximately 15.2 million people aged 50 and older were blind due to cataracts, accounting for 45% of the global blindness.^[1,2]^ In Iran, cataracts are the leading cause of low vision across all age groups, and clinically significant cataracts are present in almost one-third of the population over 54 years old.^[3,4]^ Cataract surgery has improved dramatically since Charles Kelman introduced phacoemulsification (often referred to as phaco) as an alternative to extracapsular cataract extraction (ECCE). The phacoemulsification technique has rapidly become the preferred method for cataract surgery in Iran, increasing from < 20% of all cataract surgeries in 2003 to > 95% in 2010.^[5,6,7]^ The phaco method has improved visual outcomes and dramatically decreased the prevalence of intra- and postoperative complications.^[8,9]^ Reported intraoperative complications associated with phaco include posterior capsule rupture (1.5–3.5%), iris injury (0.1–1.2%), nucleus drop into the vitreous (0.1–0.2%), zonular dehiscence (0.46%), and suprachoroidal hemorrhage (0.07%). The most common postoperative complications include posterior capsular opacification (4.2%), corneal edema (0.03–5.18%), cystoid macular edema (1.2–3.5%), wound dehiscence (0.06–1.1%), and residues of lens particles (0.45–1.7%).^[10,11,12]^

There is a learning curve associated with the phaco technique, which requires years of training to develop the necessary spatial coordination and precision of movements. In contrast to past residency programs that began with manual ECCE and gradually introduced phacoemulsification, ophthalmology residents now predominantly learn phacoemulsification as the preferred technique for cataract surgery.

Learning curve studies to date have demonstrated an inverse relationship between the number of surgeries performed by residents and the rates of intraoperative complications.^[13,14]^ Several studies have explored the average number of phacoemulsification surgeries that residents need to perform to achieve a minimum level of competency.^[15,16]^ The Accreditation Council for Graduate Medical Education mandates that trainees perform at least 86 cataract surgeries during their residency.^[17]^ The reported complication rate for resident-performed phaco ranges from 3.7% to 7.9%.^[18,19,20]^

This study aimed to determine the incidence of posterior capsule tears and identify risk factors associated with intraoperative complications in resident-performed phacoemulsification surgeries at a tertiary care academic center.

##  METHODS

The Institutional Review Board of Shahid Beheshti University's Human Subjects Research Committee approved this study (Ir.sbmu.msp.rec.1398.254). Informed consent was not required for this retrospective case series. Our research adhered to the tenets of the Declaration of Helsinki and the regulations of the Health Insurance Portability and Accountability Act. We identified all patients who underwent cataract surgery by residents between March 2020 and March 2021 at Torfeh Eye Hospital, Tehran, Iran.

Only patients with age-related cataracts who underwent the phacoemulsification procedure by residents were included. We designed a questionnaire that included patients' demographic information, systemic and ophthalmic conditions, biometric data, details of the surgery, and the training level of the residents who performed the surgery. The severity of the cataract was based on the LOCS III classification. Pupil diameter was measured 30 minutes after administering three drops of tropicamide 1% (Mydrax, Sina Daru, Tehran, Iran), given 5 minutes apart. A pupil was considered small if its diameter was < 5 mm. An anterior chamber depth of < 2.5 mm was considered shallow, and an axial length of > 26 mm was defined as high myopia. In the control group, patients without complications were selected randomly, and their information was recorded. Finally, all the data from patients with and without complications were entered in SPSS version 25 (IBM Corp., Armonk, NY, USA) for analysis.

### Cataract Surgery Training Program

At Torfeh Eye Hospital, the phacoemulsification training program is primarily conducted in the Cornea and Glaucoma services and begins in the second year of residency. During this time, residents enter the operating room and learn the essential steps of phacoemulsification using an eye surgery simulator. Additionally, they observe surgeries performed by attending physicians or senior residents. Residents are involved in their first operations as assistant surgeons, participating in steps such as wound incision and capsulorrhexis under the direct supervision of an attending surgeon. Attending surgeons may take over the procedure at any point if necessary.Third- and fourth-year residents are qualified to perform cataract surgeries as primary surgeons but still require direct supervision by attending physicians. The primary surgical techniques taught in our department are “divide-and-conquer” and “stop-and-chop.” All surgeries are performed using the Alcon Infiniti phacoemulsification system (Alcon, Fort Worth, Texas, USA). All surgeries are recorded and displayed on a monitor.

**Table 1:** Rate of intraoperative complications

**Complication**	***N*** ** (%)**
Nucleus drop	8 (0.7)
IOL drop	0 (0)
PCR + VL	29 (2.6)
PCR	2 (0.2)
Anterior capsule tear	21 (1.9)
Zonular dehiscence	3 (0.3)
Total	63 (5.6)
IOL, intraocular lens; PCR, posterior capsule rupture; VL, vitreous loss; N, number	

**Table 2:** Rate of intraoperative complications by age

**Complication**	**Age group (yrs)**					***P*** -value ${}^{*}$
	**40–49**	**50–59**	**60–69**	**70–79**	**80–89**	
	***N*** ** (%)**	***N*** ** (%)**	***N*** ** (%)**	***N*** ** (%)**	***N*** ** (%)**	
Nucleus drop	1 (0.1)	1 (0.1)	0 (0)	5 (0.4)	1 (0.1)	0.061
IOL drop	0 (0)	0 (0)	0 (0)	0 (0)	0 (0)	NA
PCR + VL	1 (0.1)	2 (0.2)	12 (1.1)	7 (0.6)	7 (0.6)	0.091
PCR	2 (0.2)	0 (0)	0 (0)	0 (0)	0 (0)	0.008
Anterior capsule tear	2 (0.2)	7 (0.6)	6 (0.5)	3 (0.3)	3 (0.3)	0.1
Zonular dehiscence	0 (0)	0 (0)	0 (0)	1 (0.1)	2 (0.2)	0.364
Total	6 (0.6)	10 (0.9)	18 (1.6)	16 (1.4)	13 (1.2)	
IOL, intraocular lens; PCR, posterior capsule rupture; VL, vitreous loss; yrs, years; N, number NA, not applicable ${}^{*}$ Based on Fisher's exact test						

**Table 3:** Rate of intraoperative complications by sex

**Complication**	**Sex**		***P*** -value ${}^{*}$
	**Male**	**Female**	
	***N*** ** (%)** * *	***N*** ** (%)** * *	
Nucleus drop	6 (0.5)	2 (0.2)	0.27
IOL drop	0 (0)	0 (0)	NA
PCR + VL	15 (1.3)	14 (1.2)	0.803
PCR	1 (0.1)	1 (0.1)	> 0.999
Anterior capsule tear	10 (0.9)	11 (1.0)	0.594
Zonular dehiscence	2 (0.2)	1 (0.1)	> 0.999
Total	34 (3.0)	29 (2.6)	
IOL, intraocular lens; PCR, posterior capsule rupture; VL, vitreous loss; NA, not applicable; N, number ${}^{*}$ Based on Fisher's exact test			

**Table 4:** Rate of intraoperative complications by type of anesthesia

**Complication**	**Type of anesthesia**		***P*** -value ${}^{*}$
	**General**	**Monitored anesthesia care**	
	***N*** ** (%)** * *	***N*** ** (%)** * *	
Nucleus drop	7 (0.6)	1 (0.1)	0.004
IOL drop	0 (0)	0 (0)	NA
PCR + VL	7 (0.6)	22 (2.0)	0.042
PCR	1 (0.1)	1 (0.1)	> 0.999
Anterior capsule tear	9 (0.8)	12 (1.1)	0.595
Zonular dehiscence	0 (0)	3 (0.3)	0.281
Total	24 (2.1)	39 (3.6)	
IOL, intraocular lens; PCR, posterior capsule rupture; VL, vitreous loss; NA, not applicable; N, number ${}^{*}$ Based on Fisher's Exact test			

**Table 5:** Rate of intraoperative complications by resident year

**Complication**	**Resident year**		***P*** -value ${}^{*}$
	**Third**	**Fourth**	
	***N (%)***	***N (%)***	
Nucleus drop	4 (0.4)	4 (0.4)	> 0.999
IOL drop	0 (0)	0 (0)	NA
PCR + VL	24 (2.1)	5 (0.4)	0.08
PCR	0 (0)	2 (0.2)	0.5
Anterior capsule tear	12 (1.1)	9 (0.8)	0.71
Zonular dehiscence	3 (0.3)	0 (0)	0.21
Total	43 (3.9)	20 (1.8)	
IOL, intraocular lens; PCR, posterior capsule rupture; VL, vitreous loss; NA, not applicable; N, number ${}^{*}$ Based on Fisher's Exact test			

**Table 6:** Univariate analysis of the association between intraoperative complications and clinical characteristics

	**Univariate**		
**Variable**	**OR**	***P*** **-value**	**95% CI**
Mean age	0.765	0.995	(0.665, 1.027)
Sex	1.221	0.537	(0.649, 2.295)
Resident year	2.238	0.017	(1.157, 4.328)
Mature/Brunescent cataract	3.096	0.032	(0.811, 5.822)
Small pupil size	4.159	0.068	(0.899, 6.249)
DM	1.774	0.086	(0.922, 3.413)
HTN	0.797	0.482	(0.722, 1.502)
PXF	2.247	0.049	(0.062, 3.955)
Phacodonesis	–	1.000	–
Corneal opacity	–	1.000	–
Shallow AC	6.889	0.069	(0.861, 7.193)
High myopia	0.626	0.742	(0.038, 10.195)
Anesthesia	1.249	0.507	(0.647, 2.411)
AC, anterior chamber; CI, confidence interval; DM, diabetes mellitus; HTN, hypertension; OR, odds ratio; PXF, pseudoexfoliation			

**Table 7:** Multivariate analysis of the association between intraoperative complications and clinical characteristics

	**Multivariate**		
**Variable**	**OR**	***P*** **-value**	**95% CI**
Resident year	3.238	0.005	(1.419, 7.389)
Mature/Brunescent Cataract	4.046	0.009	(1.005, 5.461)
Small pupil size	6.646	0.074	(0.832, 7.079)
DM	0.991	0.983	(0.452, 2.174)
Shallow AC	3.499	0.255	(0.405, 7.211)
AC, anterior chamber; CI, confidence interval; DM, diabetes mellitus; OR, odds ratio			

### Statistical Analysis

Statistical analyses were performed using SPSS version 25. Means, ranges, frequencies, and percentages were used to present the data. The *t*-test and Fisher's exact test were used to compare the results between the two groups. The data were analyzed using odds ratios derived from both univariate and multivariate logistic regression. A *P*-value of < 0.05 was considered statistically significant.

##  RESULTS

We enrolled 1121 phacoemulsification surgeries performed by third- and fourth-year ophthalmology residents between March 2020 and March 2021. Fifteen residents with a mean age of 29 ± 4 years (range, 27 to 33 years) performed the operations. All residents encountered some complications, with 2 to 7 complicated surgeries for each resident. Overall, 63 eyes (5.6%) experienced complications, including posterior capsular rupture with vitreous loss (29 eyes, 2.6%), anterior capsular rupture (1.9%), nucleus drop (0.7%), zonular dehiscence (0.3%), and posterior capsular rupture without vitreous loss (0.2%) [Table 1]. Anterior vitrectomy was performed in all cases of posterior capsular rupture with vitreous loss to clear the anterior chamber.

The mean age of the study participants was 67 ± 12 years in the complicated group and 67 ± 9 years in the control group (*P* > 0.5). There was no difference in the complication rates based on patients' age, gender, or type of anesthesia (general anesthesia versus monitored anesthesia care) [Tables 2–4].

The relationship between residents' training level and the rate of complications is shown in Table 5. Third-year residents experienced 43 cases of complications, compared to 20 cases by fourth-year residents. Furthermore, 24 out of 29 cases of posterior capsular rupture occurred with third-year residents, while 5 cases occurred with fourth-year residents. Regarding nucleus drop, an equal number of cases (four each) occurred with both third- and fourth-year residents [Table 5].

Based on univariate analysis, the main risk factors for were mature or brunescent cataract (OR = 3.096, *P *= 0.031), the resident's training year (OR = 2.238, *P* = 0.017), and the presence of pseudoexfoliation (OR = 2.247, *P* = 0.049) [Table 6].

According to the results of multivariate analysis, mature or brunescent cataracts and lower training levels among residents were found to be associated with higher complication rates [Table 7].

##  DISCUSSION

In the current study at a tertiary training center, we found a higher rate of intraoperative complications during phacoemulsification for cataract surgery in cases with mature or brunescent cataracts and when performed by less experienced surgeons. Our findings suggest that faculty supervision is a crucial factor in reducing the rate of complications in cataract surgeries performed by residents. While in some centers, residents begin performing cataract surgery in their second year of residency, at our center, they start by learning the essential steps of phacoemulsification using an eye surgery simulator and by observing surgeries performed by attending physicians or senior residents. They then participate in their first surgeries as assistant surgeons, performing steps such as wound incision and capsulorrhexis under the direct supervision of an attending surgeon. By the end of the second year, most residents are adequately qualified to perform cataract surgery as primary surgeons but still require direct supervision by senior residents or attending physicians. Moreover, faculty members carefully oversee case selection for beginning residents to ensure that they are not given complex cases to operate, such as patients with corneal opacities, miotic pupils, pseudoexfoliation syndrome, dense and mature cataracts, and diabetic retinopathy. Patients who are at high risk for general anesthesia and prefer surgery under local or topical anesthesia are also typically excluded from cases assigned to beginning residents.

Our findings align with previous studies reporting a higher rate of complications for mature cataracts^[19,20,21]^ and a lower level of training among residents.^[20,22]^ Another study analyzed 396 patients who had undergone cataract surgery performed by four residents. The residents' first 50 cases were compared with their last 50 cases, which were performed at the end of their training. They reported intraoperative complications including posterior capsular rupture without vitreous loss (0.8%) and with vitreous loss (1.3%), iris injury (0.5%), and zonular dehiscence (0.5%). The rate of intraoperative complications was 2.6% in the residents' early cases, but it decreased to 0% in their later cases. This considerable difference can be due to the small number of residents in that study. The authors concluded that proper training, supervision, and skill improvement could result in fewer complications.^[23]^ Another study on 300 phaco procedures found that the prevalence of posterior capsular rupture with vitreous loss was 4.6% in surgeries performed by second-year residents and 1.3% in surgeries performed by third-year residents.^[24]^ Woodfield et al^[19]^ investigated 691 phaco surgeries performed by second- or third-year residents and found intraoperative complication rates of 7.9% and 7.6%, respectively, with no significant difference between the two groups.^[19]^ However, the prevalence of vitreous loss was higher in surgeries performed by second-year residents (4.8%) than third-year residents (3%). Briszi et al^[20]^ reported an overall intraoperative complication rate of 3.8% in phaco procedures performed by residents. Hashemi et al^[22]^ reviewed 500 phaco surgeries by residents and reported a 10.2% rate of vitreous loss. Factors like anterior capsular rupture, prolonged effective phaco time, and unsupervised surgeries were associated with a higher risk of vitreous loss in multivariate analysis. In another study, Zare et al^[21]^ reviewed 767 resident-performed phaco surgeries and reported 7.9% cases of posterior capsular rupture and vitreous loss, which were five times more likely in surgeries performed by residents compared to those by anterior segment fellows. Factors such as older age, female gender, small pupil, small capsulorrhexis, pseudoexfoliation, and high myopia were significantly associated with vitreous loss.

In our study on 1121 patients, the rate of intraoperative complications was 5.6%. The most common complication was posterior capsular rupture with vitreous loss (2.6%), which is consistent with previous studies. This complication is the most frequent intraoperative issue with the phaco method and can lead to decreased postoperative best-corrected visual acuity, an increased risk of macular edema, retinal detachment, and endophthalmitis.^[25,26,27,28]^ Nucleus drop occurred in 0.7% of procedures, a rate which is comparable to previous reports.^[19,20,21][22][29]^

Factors associated with a higher rate of complications in our study included mature cataracts (OR = 3.096, *P* = 0.032), the training level of residents (OR = 2.238, *P* = 0.017), and pseudoexfoliation (OR = 2.247, *P* = 0.049). We found no association between intraoperative complications and other previously reported risk factors, such as small pupils, shallow anterior chambers, corneal haziness, and high myopia.^[18,30,31]^ Also, systemic diseases such as diabetes, hypertension, the patient's age, gender, and type of anesthesia did not correlate with the rate of complications. Of the 63 cases with complications, 39 underwent monitored anesthesia care.

Our study was a retrospective analysis conducted at a single center, which may limit the generalizability of our findings to the larger population of residents. Another limitation is the small sample size, as well as the fact that complex cases were exclusively managed by attending surgeons. This may have prevented us from identifying statistically significant differences for some known risk factors for vitreous loss, as reported in larger studies. We recommend conducting further multicenter prospective studies to accurately determine the risk factors for vitreous complications in cataract surgeries performed by residents.

In summary, we observed a higher rate of intraoperative complications in surgeries performed by less experienced residents and in cases of mature and brunescent cataract. According to the results of this study, phacoemulsification cataract surgery for patients with white and brunescent mature cataracts should be performed only by fourth-year residents. Appropriate case selection and direct supervision by more experienced surgeons could decrease the risk of intraoperative complications and improve the visual outcomes.

##  Financial Support and Sponsorship

None.

##  Conflicts of Interest

None.
